# Introduction of the generic sense of ability to adapt scale and validation in a sample of outpatient adults with mental health problems

**DOI:** 10.3389/fpsyg.2023.985408

**Published:** 2023-03-29

**Authors:** Katinka Franken, Pauline Schuffelen, Peter ten Klooster, Karin van Doesum, Gerben Westerhof, Ernst Bohlmeijer

**Affiliations:** ^1^Department of Psychology, Health and Technology, Faculty of Behavioural, Management and Social Sciences, University of Twente, Enschede, Netherlands; ^2^GGNet Centre of Mental Health, Apeldoorn, Netherlands; ^3^Mindfit, Deventer, Netherlands

**Keywords:** scale development, positive psychology assessment, reliability, validation, well-being, adaptation, generic sense of ability to adapt scale, mental health problems

## Abstract

**Introduction:**

The ability to adapt is a core aspect of daily human life. Recent models and theories emphasize its essential role for health and well-being. It concerns the perceived ability to readjust and actively deal with the psychosocial consequences of challenging events. While many questionnaires measure competences related to adaptability to specific conditions, a scale that measures a generic sense of the ability to adapt is lacking. The aim of the present study is to introduce the Generic Sense of Ability to Adapt Scale (GSAAS) and to examine its psychometric properties.

**Methods:**

The article describes two sub-studies. In the first study the items of the GSAAS were generated and field-tested in a cross-sectional non-clinical sample using item analysis, exploratory factor analysis and Rasch analysis.

**Results:**

This resulted in a 10-item questionnaire measuring a single dimension with good reliability (Cronbach’s *α* = 0.87). In the second study the 10-item scale was validated using a cross-sectional sample of 496 outpatient adults with mental health problems. Confirmatory factor analysis confirmed the unidimensional structure of the GSAAS and the absence of measurement variance across gender, age and education. Reliability was high (*α* = 0.89) and moderate to strong correlations between the GSAAS and concurrent validation measures confirmed its convergent validity. Regarding incremental validity, the GSAAS accounted for 7.4% additional explained variance in symptomatic distress above and beyond sense of coherence.

**Discussion:**

In conclusion, the GSAAS appears to be a reliable and valid instrument to assess people’s generic sense of the ability to adapt. It is a practical and quick tool that can be used to measure a vital aspect of health in research and clinical treatment settings.

## Introduction

The ability to adapt is a core aspect of daily human life. People need to adapt to both daily hassles and major life-events in private or working conditions including accidents, chronic illnesses, and trauma in order to obtain or retain quality of life (Taylor, 1983; Lu, 1991; Helgeson, 1999; Livneh, 2001; Perrig-Chiello et al., 2015). However, a generic comprehensive measure of perceived ability to adapt is not yet available. To address this, the current study presents the development and psychometric evaluation of the Generic Sense of Ability to Adapt Scale (GSAAS).

Over the years, various theories and models of adaptation have been developed such as Taylor’s cognitive adaptation theory and Roy’s adaptation model. The cognitive adaptation theory defines adaptation as an adjustment process that involves finding ways to give meaning to a threatening event, regaining a sense of mastery, and enhancing and restoring self-esteem (Taylor, 1983). Roy’s adaptation model (Roy, 2009), widely used in nursing practice, considers the individual as a biopsychosocial being that is continuously interacting with and adapting to its environment. The model discerns different adaptive modes that are both physical (physiologic needs) and psychosocial (self-concept, role function, and interdependence) (Nayback, 2009). In order to stay healthy, a person needs to be able to integrate internal stimuli (one’s thoughts and feelings) and external stimuli (the environment) aiming for the ultimate goal of achieving dignity and integrity (Nayback, 2009). Based on the analysis of 89 articles, Londono and McMillan (2015) described psychosocial adaptation as a continuous process driven by interactions with one’s self (e.g., adopting spiritual beliefs) and one’s environment (e.g., social support).

Adaptation theories have in common that adaptation serves the aim to maintain health and well-being as a dynamic equilibrium (Headey and Wearing, 1991; Antonovsky, 1996; Suh et al., 1996; Cummins, 2010). Dodge et al. (2012) define well-being as the balance point between the challenges that a person is facing and its resources. In order to maintain an acceptable level of well-being, individuals should regulate their emotions and modify their actions and beliefs (Kashdan and Rottenberg, 2010; Audulv et al., 2016). Kloep et al. (2009, p. 337) describe the adaptation process as follows: “Each time an individual meets a challenge, the system of challenges and resources comes into a state of imbalance, as the individual is forced to adapt his or her resources to meet this particular challenge.”

Based on these theories of adaptation, well-being, and dynamic equilibrium, Bohlmeijer and Westerhof (2020, 2021) developed a model of sustainable mental health, in which the ability to adapt has a central place. The model asserts that a person will continuously experience minor or major life-events challenging one’s ability to adapt. Successful adaptation will result in the absence of severe distress and the presence of mental well-being (Gross and John, 2003; Aldao et al., 2010). The ability to adapt is positively influenced by personal and contextual resources such as optimism (Schiavon, et al.,2017) and high-quality relationships (Amati et al., 2018; Van Loan and Garwood, 2020) and negatively influenced by personal and contextual barriers such as rumination (Willis and Burnett, 2016), dysfunctional emotion-regulation (Rice and Hoffman, 2014; Perry et al., 2020), and non-supportive contexts (Holeva et al., 2001).

In a similar way, Huber et al. (2011) proposed the ability to adapt as a core aspect of health. They argued that the current definition of the World Health Organization (WHO) of health as “a state of complete physical, mental and social well-being and not merely the absence of disease or infirmity” underestimates the ability of individuals with chronic illness or psychopathology to adapt to the challenges of life while maintaining life satisfaction. Rather (chronic) illness, psychopathology, and setbacks in life can be considered as challenges that disturb one’s equilibrium and require adaptation. Huber et al. (2016) developed the concept of positive health and defined it as “the ability to adapt and self-manage in the face of social, physical, and emotional challenges.” Recently, a Dutch Delphi study resulted in consensus on a similar definition of mental health as “the way you relate to yourself (personal component) and to others (social component) and how you deal with the challenges in everyday life (functional component) taking into account how you and others in society experience this (perceptive component)” (Van Bon-Martens et al., 2022).

Given its vital role in recent models of (mental) health and well-being, developing a reliable and valid measure of the ability to adapt is an important aim (Jambroes et al., 2016). Prinsen and Terwee (2019) recently tried to develop an outcome instrument to measure the domains and aspects of Huber’s concept of positive health. However, a content validity study to evaluate a preliminary 46-item questionnaire showed major concerns about its relevance, comprehensiveness, and comprehensibility. Further refinement of the conceptualization of positive health and the ability to adapt is thus warranted. In the past, various reliable and valid measures have been developed to measure *specific skills and competences* that promote the ability to adapt such as psychological flexibility (Brown and Ryan, 2003; McCracken and Yang, 2006; Vowles et al., 2008; Wicksell et al., 2010; McCracken et al., 2015; Yu et al., 2016, 2017; Terhorst et al., 2020), adaptive coping (Chesney et al., 2006; Greenaway et al., 2015; González-López et al., 2022), and optimism and cognitive styles (Conversano et al., 2010; Schiavon et al., 2017). However, a reliable and valid generic comprehensive measure of perceived ability to adapt more focused on the process than on skills, is still lacking. Therefore, we aimed to develop an instrument that captures a person’s trust to successfully cope with the psychosocial consequences of smaller or larger challenges in life.

Based on the described theoretical frameworks, it is relevant to arrive at a definition of the sense of ability to adapt, which will be the starting point for the development of a generic instrument. The focus on self-management as a *skill* to deal with chronic diseases is still too limited. The promotion of health can be seen as a continuous *process* of adaptation to (negative and positive) complexities in life, both in the environment and in the human being himself. In conclusion, we defined a generic sense of the ability to adapt as the perceived ability to readjust and actively deal with the psychosocial consequences of challenging events.

The importance of the ability to adapt for health has been the subject of research for some time. Antonovsky (1979) introduced the sense of coherence (SOC) as a potential protective factor in dealing with stress and staying healthy. SOC is a relatively stable general orientation to life and includes the ability to assess stressful life events as understandable, meaningful, and manageable (Antonovsky, 1993). An existing instrument that most closely matches the concept of the sense of the ability to adapt is the SOC-13 (Antonovsky, 1987). The external validity of the SOC with various indicators of health has been extensively studied (Morita et al., 2014; Länsimies et al., 2017). However, the items of the SOC-13 emphasize *powerlessness* and *environmental influence*. Additionally, Eriksson and colleagues indicate that focusing on health and on people’s resources is more than the measurement of the sense of coherence (Lindström and Eriksson, 2010; Eriksson and Mittelmark, 2017). With the development of the current new instrument, the emphasis on *decisiveness* and the *process of dealing with circumstances* is pursued.

The aim of the present study is twofold and elaborated in two sub-studies. The first objective, accommodated in study 1, is to introduce a new reliable and valid instrument measuring people’s generic sense of ability to adapt (GSAAS). In this first study, the items of the GSAAS were generated and field-tested in a non-clinical sample, striving to develop an instrument in which items deal with one’s trust in successful coping with (psychosocial consequences of) challenges and in shaping life, and not so much with the resources or outcome of adaptation. We expected the generic instrument to be unidimensional in nature.

The second objective, accommodated in study 2, is to validate the GSAAS in a sample of outpatient adults with mental health problems. In this second study, further validation research was carried out with the GSAAS, including confirming its factor structure, measurement invariance, reliability, and convergent and incremental validity. Besides confirming its unidimensional structure, the potential role of major characteristics of respondents as age, sex, and education was explored with data-driven multiple group measurement invariance testing. Regarding construct validity, we expect a strong association with emotional and psychological well-being. Psychological well-being is the ability to direct one’s life a meaningful way (Ryff, 1989). As this is dependent on the ability to cope with minor and major life challenges, a strong association between the two constructs is expected. In a similar vein, the absence of a sense of ability to adapt will most likely lead to an increase of feelings of powerlessness and meaninglessness and an increase of worrying. Therefore, we expect strong associations between the GSAAS and indicators of depression (Cuijpers et al., 2021) and anxiety (Roos et al., 2021) as well. Based on the earlier discussed similarities between the GSAAS and SOC-13, we also expect strong associations between these two measures. On substantive grounds, it was expected that an instrument that emphasizes the process, decisiveness, and the way of dealing with positive and negative circumstances would add unique additional variance in symptomatic distress above and beyond the sense of coherence.

## 1. Study 1: Introduction of the generic sense of ability to adapt scale

### 1.1. Materials and methods

#### 1.1.1. Phase 1: Content validity: Item generation and pre-testing

For the initial item pool generation of the intended GSAAS, we used a theoretical approach based on the aforementioned theories and models of adaptation. Study of literature about adaptation skills yielded sources for developing an item pool to ensure good content validity (Vaillant, 2000; Fredrickson, 2001; Bjorklund, 2015). Potential items for the GSAAS were produced in an iterative process by two of the authors (EB and PtK) and four psychology students. The aim was to develop an instrument measuring generic adaptation skills applicable to both the general population and to somatic and psychiatric patient groups in line with the new model of sustainable mental health, which integrates positive psychology into clinical models of mental health and endorses the importance of adaptation processes in regulating one’s well-being (Bohlmeijer and Westerhof, 2020, 2021). For this purpose, as mentioned before, we defined a generic sense of the ability to adapt as the perceived ability to readjust and actively deal with the psychosocial consequences of challenging events.

Taking this aim into account and striving for clearly formulated questions, several rounds of review took place. All 23 potential items were positively formulated and a 5-point Likert-type rating scale (not at all, hardly ever, seldom, mostly, always) was used because of its accuracy and reliability and to generate sufficient response variance (Joshi et al., 2015). To ensure the usability, readability, and understandability, the draft questions were cognitively pre-tested among five participants who were asked to verbalize their thoughts while answering the items. Think-aloud methods are a robust and flexible technique and are a proven way to optimize item-sets (Cotton and Gresty, 2006; Schulte-Mecklenbeck et al., 2011). Based on the think-aloud pre-tests, four items were removed due to insufficient clarity for the participants.

#### 1.1.2. Phase 2: Field-testing: Factor structure and item reduction

##### 1.1.2.1. Participants

After the pre-testing, the 19 remaining items were field-tested for item quality evaluation and item reduction purposes in an online survey study among a convenience sample of 250 participants, consisting mostly of students, aged 18–75 years (*M* = 24.7, *SD* = 10.7). Most respondents were female, highly educated, living with friends, parents, or a partner and one-fifth had a paid job. Two-third were Dutch and one third had the German nationality (see Table 1). All participants provided complete and valid responses.

**Table 1:** Major characteristics of respondents.

		Study 1 total (*N* = 250)	(%)	Study 2 total (*N* = 496)	(%)
Gender *n* (%)					
	Male	67	(26.8)	187	(37.9)
	Female	183	(73.2)	308	(62.1)
Age					
	Mean	24.7		36.7	
	Range	18–75		17–75	
	*SD*	10.7		14.4	
Level of education *n* (%)^a^					
	Low	2	(0.8)	59	(12.3)
	Moderate	44	(26.0)	240	(50.0)
	High	194	(81.6)	181	(37.7)
Marital status *n* (%)					
	Single without children	23	(9.2)		
	Single with children	0	(0.0)		
	Married without children	49	(19.6)		
	Married with children	20	(8.0)		
	Living with parents	59	(23.6)		
	Living with friends	95	(38.0)		
	Other	4	(1.6)		
Nationality					
	Dutch	167	(66.8)		
	German	83	(33.2)		
Daily activities *n* (%)					
	School	192	(76.8)		
	Volunteer/home work	7	(2.8)		
	Work	47	(18.8)		
	Sick at home	2	(0.8)		
	Unemployed	2	(0.8)		

^a^low = primary school, lower vocational education; moderate = secondary school, intermediate vocational education; high = higher vocational education, university.

##### 1.1.2.2. Procedure

The survey study was approved by the ethical committee of the University of Twente (reference number: 15092). Data collection was performed in the first quarter of 2015 by a group of second-year psychology students. Participants were recruited on a voluntary basis through Sona (the university’s system for obtaining mandatory research credits for first-and second-year psychology and communication students) and the students’ personal networks. Data were collected in the Dutch language.

##### 1.1.2.3. Data analysis

Item analysis and selection followed an iterative process. First, items were screened for skewness and removed when skewness exceeded the range of ±1. Next, exploratory factor analysis (EFA) was performed with the remaining items using geomin (oblique) rotation in Mplus 7.11 (Muthén and Muthén, 1998). Given the ordinal nature of the items, robust maximum likelihood estimation was used. The number of factors underlying the items was determined using both the screen test (Cattell, 1966) and parallel analysis (Horn, 1965) with 50 random data sets. After this EFA, a Rasch analysis was done using the partial credit model in Winsteps 3.65 (Linacre, 1991) to further examine the unidimensionality of the items and their fit to the Rasch model (Bond et al., 2007). Items with an infit and outfit means square statistic between 0.7 and 1.3 were considered to show sufficient fit (Wright, 1994). A second Rasch analysis was performed using only those items demonstrating adequate fit. Finally, based on the content of remaining items, a last selection was made. A new Rasch analysis was done and a final set of items was adopted, which was further administered and examined in study 2.

At different stages, internal consistency of the item set (Cronbach’s alpha) was computed by using the IBM Statistical Package for Social Sciences (SPSS) version 27. 95% confidence intervals (Cis) for Cronbach’s alpha estimates were calculated by bootstrapping 1,000 times. Guidelines by Kline (2000) were applied: values of Cronbach’s alpha below 0.70 were interpreted as low and unacceptable, an alpha between 0.70 and 0.79 fair, an alpha between 0.80 and 0.89 good and an alpha above 0.90 excellent.

### 1.2. Results

**Figure 1:**
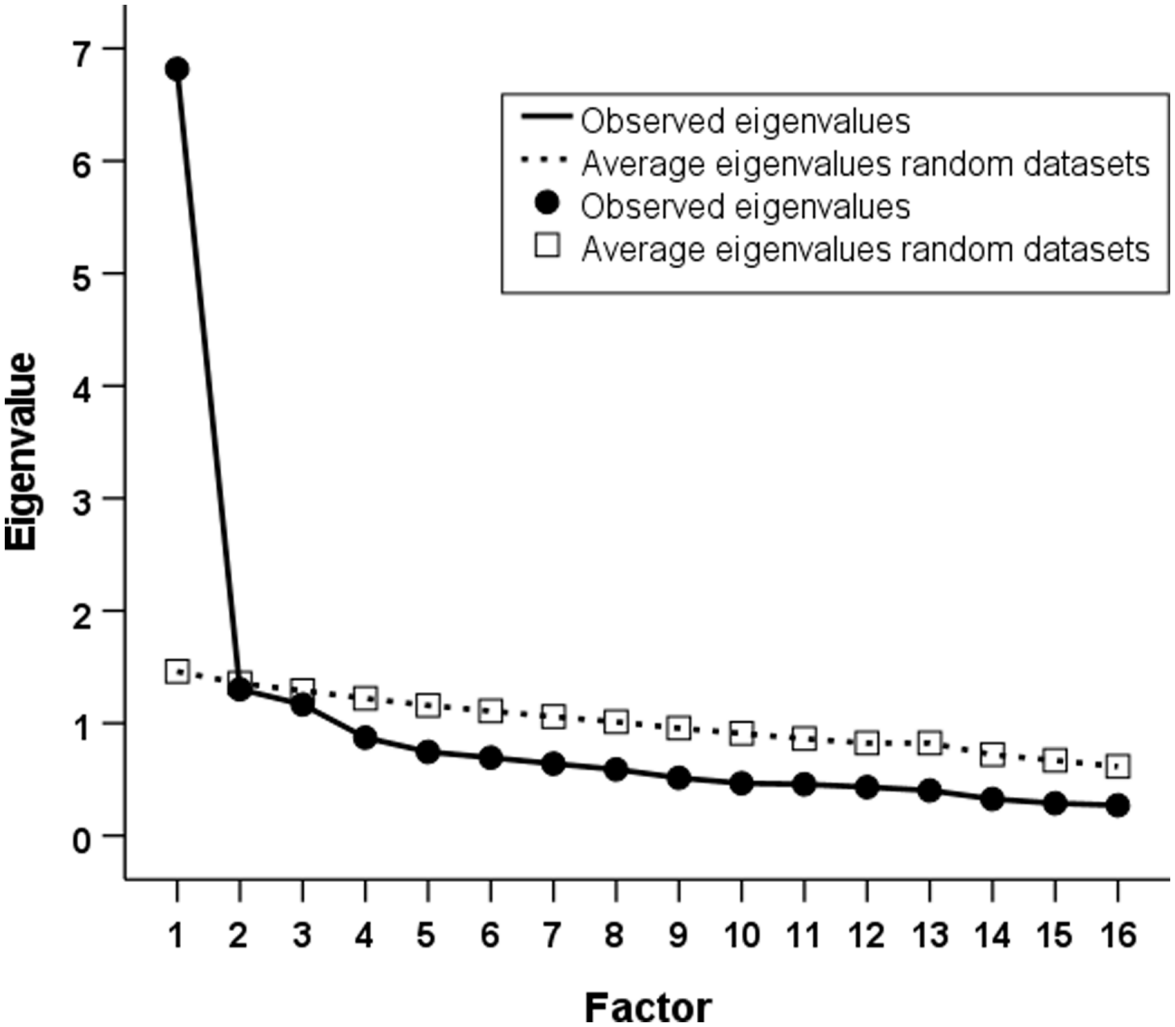
Scree plot of observed eigenvalues and average eigenvalues from parallel analysis (50 random datasets).

Three of the 19 items demonstrated skewness values greater than 1. EFA with the 16 remaining items resulted in three factors with eigenvalues >1. However, the scree plot indicated a clear single-factor solution (see Figure 1). The eigenvalue of the first factor was 6.819 whereas it was 1.303 and 1.168 for the second and third factor, respectively. The parallel test confirmed this single-factor solution by showing that only the first factor in the real data had an eigenvalue higher than its counterpart average eigenvalue from the random data (average random eigenvalue = 1.461). All items loaded strongly on this first factor, with factor loadings ranging from 0.465 to 0.711. Internal consistency of the 16 items was excellent with Cronbach’s *α* = 0.907 (95% CI: 0.890–0.929).

To further examine the fit of the items to a unidimensional model, a Rasch analysis followed the EFA. Two items showed a poor fit to the Rasch model, with, respectively, outfit mean square statistic of 1.37 and 1.48, and being removed. A 14-item scale remained that showed no remaining misfitting items in subsequent Rasch analysis (infit mean square statistics: *M* = 1.00, range 0.82–1.28; outfit mean square statistics: *M* = 1.00, range 0.82–1.22).

Based on the content of these 14 items, a final selection was made on substantive considerations. To guarantee optimal applicability, also by people in non-clinical populations, we decided to remove the four items focusing on the *skill* of self-management. These four items specifically addressed dealing with *diseases*, whereas the other items are more focused on the *process* of dealing with all kinds of complexities in life. Given that psychosocial adaptation was defined as a process by which a person interacts with the psychosocial consequences of smaller or larger challenges in life (e.g., daily hassles, chronic and/or severe physical diseases, life-events such as divorce, accidents, and job-losses) and goes beyond coping with illness, we decided to not include them in the final instrument.

A new Rasch analysis was done with the 10 remaining items, which showed no substantial consequence of the removal of these items for the measurement range and precision of the scale. There was only marginal misfit for one item (infit mean square statistic = 1.31), which also had a lower factor loading (0.487) in the initial factor analysis. A final Rasch analysis showed that removing or retaining this item would not have consequences for either measurement range or measurement precision (see Table 2). Based on its content relevance (“*I have influence over my personal circumstances*”), this item was retained in the scale. The final 10 items (see Table 3) demonstrated a reasonable spread of item difficulty levels across the scale. Internal consistency of these 10 items was good: Cronbach’s *α* = 0.872 (95% CI: 0.847–0.897).

**Table 2:** Rasch analysis results of the final 10 items of the GSAAS.

Item	Item difficulty in logits (SE)	Infit MNSQ	Outfit MNSQ
I can cope well with adverse circumstances	0.33 (0.10)	0.82	0.83
I feel energetic	−0.62 (0.11)	0.95	1.01
I see plenty of interesting challenges	−0.52 (0.11)	1.06	1.05
I can cope well with the stress in my life	0.63 (0.10)	0.92	0.93
I have influence over my personal circumstances	−0.19 (0.11)	1.31	1.20
I can easily handle setbacks	−0.16 (0.10)	0.82	0.81
If something unexpected happens, I can easily adapt	0.26 (0.10)	1.08	1.08
I can cope well with my daily life	−0.91 (0.12)	0.92	0.94
If I encounter difficulties, I can find a way out	1.06 (0.11)	0.88	0.88
If I want something, I go for it	0.11 (0.11)	1.19	1.23

SE, standard error; Infit, information-weighted fit; MNSQ, mean square; Outfit, outlier-sensitive fit.

**Table 3:** Completely standardized factor loadings for the 10 item one-factor solution of the GSAAS.

Item	Standardized factor loadings
I can cope well with adverse circumstances	0.758
I feel energetic	0.710
I see plenty of interesting challenges	0.588
I can cope well with the stress in my life	0.733
I have influence over my personal circumstances	0.544
I can easily handle setbacks	0.600
If something unexpected happens, I can easily adapt	0.713
I can cope well with my daily life	0.640
If I encounter difficulties, I can find a way out	0.762
If I want something, I go for it	0.617

The final set of 10 items was further investigated in study 2 to test the reliability and validity of the GSAAS in a clinical population.

## 2. Study 2: The validation of the GSAAS in a clinical population

### 2.1. Materials and methods

#### 2.1.1. Participants

The psychometric validation involved 496 Dutch adults (62.1% females) with a Diagnostic and Statistical Manual of Mental Disorders (DSM-5) classification (American Psychiatric Association, 2013), aged 17–75 (*M* = 36.7; SD = 14.37), and with educational levels ranging between low (12.3%), intermediate (50%), and high (37.7%) (see Table 1). All participants completed questionnaires (see 2.1.3.) prior to their intake and were referred by their general practitioner, based on its estimated mild-to-moderate DSM-5 classification, to receive outpatient treatment in primary mental health care at MindFit Mental Health Centre. In comparison with normative OQ-45 scores from outpatients from three Dutch public mental health care organizations (De Jong et al., 2007), current Symptomatic Distress scale scores (*M* = 46.7; SD = 20) and Social Role scale scores (*M* = 12.5; SD = 5) were similar. The total Psychopathology score was considerably lower in the current population (*M* = 50.5; SD = 20), primarily due to a lower average Interpersonal Relations scale score (*M* = 10.5; SD = 5). Each registered patient at MindFit during July 2018 and March 2019 was asked to participate on a voluntary basis in this second study and no incentives were given.

#### 2.1.2. Procedure

Study 2 was evaluated by The Medical Ethics Review Committee (METC) from Twente, which declared that the current study was not subject to ethical review under the Law on Research Involving Human Subjects (file number: K18-19). MindFit’s Routine Outcome Monitoring (ROM) system was used to digitally collect data. As part of a larger cross-sectional study on sustainable mental health, participants filled out seven different self-report measures before their intake interview of which we used four in the present study. Participants provided their informed consent for the use of data for scientific purposes. Participants were included from 18 years, on the terms that they had a valid email-address, access to internet and a proficiency in Dutch.

#### 2.1.3. Measures

##### 2.1.3.1. Ability To adapt

The Generic Sense of Ability to Adapt Scale (GSAAS) is the instrument under evaluation in this study. It is a 10-item scale measuring the extent to which an individual feels to be able to readjust and actively deal with the psychosocial consequences of personally challenging events (see Table 3). All items were scored on a 5-point Likert scale, which ranged from *not at all* (0) to *always* (4). Included items are for example “*I see plenty of interesting challenges*” and “*If something unexpected happens, I can easily adapt*.” A higher total average score reflects a higher generic sense of ability to adapt and to adjust to daily life challenges and adverse life-events, whereas low scores suggest low perceived ability to adapt.

To measure convergent and incremental validity, the scales mentioned below were used.

##### 2.1.3.2. Sense of coherence

Sense Of Coherence (SOC) was measured using Antonovsky’s SOC-13, also known as the Orientation to Life Questionnaire. The SOC-13 comprises 13 items with three subscales (Antonovsky, 1987). The first subscale is *comprehensibility,* measuring the extent to which stimuli are considered predictable, structured, explicable, and understandable. The second subscale is *meaningfulness,* which measures the extent to which someone experiences adversities as challenges with emotional meaning and valuable to adapt to. The third subscale is *manageability,* measuring the availability of resources to deal with circumstances and demands (Mittelmark et al., 2016). All items were scored on a 7-point semantic differential scale with two opposite response options (e.g., never to very often) tailored to each question. Five of the items were reversed (1, 2, 3, 7, 10) so a higher mean reflected a higher level of the SOC. Although the scale consists of three subscales, Antonovsky (1987) intended to measure SOC as a general factor. Various studies evaluating the SOC scale support this idea of SOC as an overarching factor with three dimensions (Klepp et al., 2007; Drageset and Haugan, 2016; Mittelmark et al., 2016). In the present study the scale proved to have a good internal consistency (Cronbach’s α = 0.871, 95% CI: 0.835–0.896).

##### 2.1.3.3. Well-being

Well-being was measured using the Mental Health Continuum-Short Form (MHC-SF). The MHC-SF is designed to measure positive mental health and consists of 14 items divided into three subscales (Keyes et al., 2008). The first subscale is E*motional Well-being* (EW), which measures positive and negative affect and life-satisfaction. The second subscale is S*ocial Well-being* (SW), measuring someone’s functioning in society. The third subscale is P*sychological Well-being* (PW), reflecting positive functioning in the individual’s life. On all items, participants were asked to rate how frequently they have experienced a given feeling during the past month. Items were answered on a 6-point Likert scale which ranged from *never* (0) to *every day* (5). Studies evaluating psychometric properties of the Dutch MHC-SF show its reliability and validity (Lamers et al., 2011; De Vos et al., 2017; Franken et al., 2018; De Beurs et al., 2022). Within the sample of the current study, the subscales and total scale proved to have fair to excellent internal consistency in the present study (EW Cronbach’s *α* = 0.886, 95% CI: 0.864–0.903; SW *α* = 0.799, 95% CI: 0.764–0.826; PW *α* = 0.855, 95% CI: 0.833–0.873; total scale *α* = 0.920, 95% CI: 0.908–0.930).

##### 2.1.3.4. Psychopathology

Psychopathology was measured using the Outcome Questionnaire 45 (OQ-45) (De Jong et al., 2007). The OQ-45 consists of 45 items comprising three subscales. The first subscale is *Symptomatic Distress* (OQ-SD), consisting of items with the most common symptoms in psychopathology (e.g., anxiety and depression). The second subscale is *Interpersonal Relations* (OQ-IR), measuring the functioning of patients in relationships with family and friends. The third subscale is *Social Role* (OQ-SR) that assesses a patient’s functioning in work and leisure. Items were answered on a 5-point Likert scale which ranged from *never* (0) to *almost always* (4). Nine items were reversed so a higher mean reflects a higher level of psychological complaints. In the present study, both the OQ-SD scale (Cronbach’s *α* = 0.950, 95% CI: 0.937–0.959) and the total scale (Cronbach’s *α* = 0.925, 95% CI: 0.863–0.952) showed excellent internal consistency. The internal consistency of the OQ-IR scale (Cronbach’s *α* = 0.733, 95% CI: 0.656–0.785) and the OQ-SR scale (Cronbach’s *α* = 0.723, 95% CI: 0.662–0.770) was acceptable.

#### 2.1.4. Data analysis

SPSS version 27 was used for determining the descriptive and correlational statistics and for calculating the internal consistency of the scale (Cronbach’s alpha). Confirmatory factor analysis (CFA) was performed using Mplus 7.11 (Muthén and Muthén, 1998).

##### 2.1.4.1. Factorial validity

Confirmatory factor analysis using robust maximum likelihood estimation was applied to evaluate whether the single-factor structure of the GSAAS, found in the non-clinical sample (study 1), could be confirmed in the clinical population. The data were tested against a strict 10-item model in which all items loaded on a single latent factor. The model’s fit was determined using the Satorra-Bentler (SB) scaled Chi-square statistic (*χ*^2^) in which a small non-significant value is indicative for good fit. Because the *χ*^2^ is overly sensitive to misfit, a ratio of <5 between *χ*^2^ and the degrees of freedom was considered indicative of an acceptable fit (Watkins, 1989). In addition, the Tucker–Lewis Index (TLI), the Comparative Fit Index (CFI), the Standardized Root Mean Square Residual (SRMR), and the Root Mean Square Error of Approximation (RMSEA) were used to test the model’s fit (Hu and Bentler, 1998). An acceptable fit is achieved when TLI ≥ 0.90, CFI ≥ 0.90, SRMR ≤0.10, and RMSEA ≤0.08, while a good model fit is established when TLI ≥ 0.95, CFI ≥ 0.95, SRMR ≤0.08, and RMSEA ≤0.06 (Browne and Cudeck, 1992; Hu and Bentler, 1999). Standardized factor loadings were considered sufficient and meaningful if they exceeded 0.40 (Floyd and Widaman, 1995; Hair et al., 2006).

##### 2.1.4.2. Measurement invariance

To explore whether meaningful comparisons across different groups can be made using the GSAAS, multiple-group confirmatory factor analyses were executed to establish measurement invariance. People were grouped based on the demographics gender (male vs. female), age (<35 vs. >35 years) and educational level (low/moderate vs. high). The data were analyzed on three increasingly restrictive levels of invariance: configural invariance (same factor structure), metric invariance (same factor structure and equal factor loadings), and scalar invariance (same factor structure and equal factor loadings and intercepts). The *χ*^2^ difference test (Δ*χ*^2^) was only used for descriptive purposes because of its sensitivity to sample size (Chen, 2007). Instead, Chen (2007) suggests to use the absolute changes in CFI and RMSEA indices to determine the evidence for sufficient measurement invariance because they appear to be equally sensitive to the three types of invariance tests. Based on the current sample size (*N* > 300), a change of ≥ −0.010 in ΔCFI, supplemented by a change of ≥0.015 in ΔRMSEA or ≥0.030 in ΔSRMR, would indicate non-invariance (i.e., a different meaning of the construct to different groups) on the metric invariance level. For scalar testing a change of ≥ −0.010 in ΔCFI, supplemented by a change of Δ ≥ 0.015 in RMSEA or a change of ≥0.010 in ΔSRMR indicates non-invariance.

##### 2.1.4.3. Convergent validity

Convergent validity was explored by calculating Pearson’s correlation coefficients between the scores on the GSAAS and scores on the convergent constructs: SOC-13 (sense of coherence), MHC-SF (well-being) and OQ-45 (psychopathology). In addition, the correlation of the GSAAS and the subscales of the OQ-45 and the MHC-SF were assessed. Values between 0.50 and 1.00 were considered strong, between 0.30 and 0.50 moderate, between 0.10 and 0.30 small, and values <0.10 weak (Cohen, 1988).

The perceived ability to adapt was expected to show strong positive relations to both the sense of coherence measure and the three core components of well-being (Keyes, 1998, 2002; Keyes et al., 2008). It was expected that the presence of ability to adapt will result in higher levels of positive affect and life-satisfaction (EW) and will be related to indicators of better positive functioning such as experiencing autonomy, mastery and meaning (PW), and social embedding (SW). Londono and McMillan (2015) and van Leeuwen et al. (2012) suggest that aspects of emotional well-being are outcomes of successful adaptation. Aspects of psychological well-being, for example finding meaning, environmental mastery, and psychological functioning, are also attributed to adaptation (Taylor, 1983; Stanton et al., 2007; Helgeson et al., 2014). Moreover, social well-being can promote successful adaptation (Stanton et al., 2007; Brüssow, 2013; Londono and McMillan, 2015; Audulv et al., 2016). Consequently, a strong positive relation between the GSAAS and general well-being was expected. Additionally, in the current sample with mild-to-moderate psychopathology, the perceived ability to adapt was expected to show a strong negative relation with symptomatic distress and moderate negative relations with functioning in relations with friends or family and in work or leisure. These expectations are in line with the model of Sustainable Mental Health, stating that well-being is seen as an outcome of successful adaptation and psychopathological symptoms are negatively related to adaptation (Bohlmeijer and Westerhof, 2020, 2021).

##### 2.1.4.4. Incremental validity

Finally, using a hierarchical linear regression analysis, incremental validity of the GSAAS was examined by determining the degree of variability in symptomatic distress (measured by OQ-SD) explained by the GSAAS beyond a sense of coherence (SOC-13). A significant increase (*p* < 0.05) in explained variance was considered indicative for incremental validity of the GSAAS. In the present study, we expected that the GSAAS, measuring the generic sense of ability to adapt, can significantly explain symptomatic distress beyond the sense of coherence.

### 2.2. Results

#### 2.2.1. Factor structure, measurement invariance, and internal consistency

The strict unidimensional factor model showed an acceptable fit based on the CFI (0.91) and the SMRS (0.05). In addition, all items (*N* = 10) showed strong factor loadings (0.544–0.762; see Table 3). However, the χ^2^/df ratio (5.36), TLI (0.88) and the RMSEA (0.10, 90% CI: 0.08–0.11) indicated a poor fit to the data. Because of an unacceptable fit of these indices, it was tested whether allowance of an error correlation between items with substantive overlap would result in a better fit. After investigating the modification indices, we allowed for an error correlation between item 2 (“*I feel energetic*”) and 3 (“*I see plenty of interesting challenges*”), which improved the fit indices (χ^2^/df = 4.37, TLI = 0.91, CFI = 0.93, RMSEA = 0.08, SRMR = 0.05). The model, in which the error correlation between item 2 and 3 (*r* = 0.34) was allowed, showed a significantly better fit based on a Satorra-Bentler scaled χ^2^ difference test (Δχ^2^ (1) = 31.21, *p* < 0.001) and adequate to good fit on all indices.

Results of the measurement invariance tests supported measurement invariance on both the metric and the scalar level for all tested demographics (see Table 4). Although χ^2^ difference tests were mostly significant (as expected), none of the used models showed relevant changes on the CFI and RMSEA or SRMR indices. This indicates that scores on the GSAAS are equivalent between these subgroups (gender, age, and educational level). The scale showed good internal consistency with a Cronbach’s α value of 0.892 (95% CI: 0.875–0.907).

**Table 4:** Measurement invariance between gender, age and educational level on the GSAAS.

Group and MI level	χ^2^ (df)	CFI	RMSEA (90% CI)	SRMR	Δχ^2^(df)	*p*	ΔCFI	ΔRMSEA	ΔSRMR
Gender									
Configural	182.53 (68)	0.931	0.084 (0.069–0.099)	0.050					
Metric	208.20 (77)	0.920	0.084 (0.071–0098)	0.064	25.99 (9)	0.0021	−0.011	0.000^*^	0.014^*^
Scalar	231.82 (86)	0.912	0.084 (0.074–0.097)	0.070	23.54 (9)	0.0051	−0.008^*^	0.000^*^	0.006^*^
Age									
Configural	173.63 (68)	0.937	0.080 (0.066–0.095)	0.051					
Metric	184.07 (77)	0.936	0.076 (0.062–0.090)	0.056	8.75 (9)	0.4608	0.001^*^	−0.004^*^	0.005^*^
Scalar	226.96 (86)	0.916	0.083 (0.071–0.097)	0.066	45.62 (9)	0.0000	−0.002^*^	0.007^*^	0.010
Education									
Configural	179.69 (68)	0.931	0.083 (0.069–0.098)	0.049					
Metric	195.93 (77)	0.926	0.081 (0.067–0.095)	0.059	15.09 (9)	0.0884	−0.005^*^	−0.002^*^	0.010^*^
Scalar	219.27 (86)	0.917	0.081 (0.068–0.094)	0.064	23.39 (9)	0.0054	−0.009^*^	0.000^*^	0.005^*^

^*^Supports the absence of measurement non-invariance.

#### 2.2.2. Convergent and incremental validity

**Figure 2:**
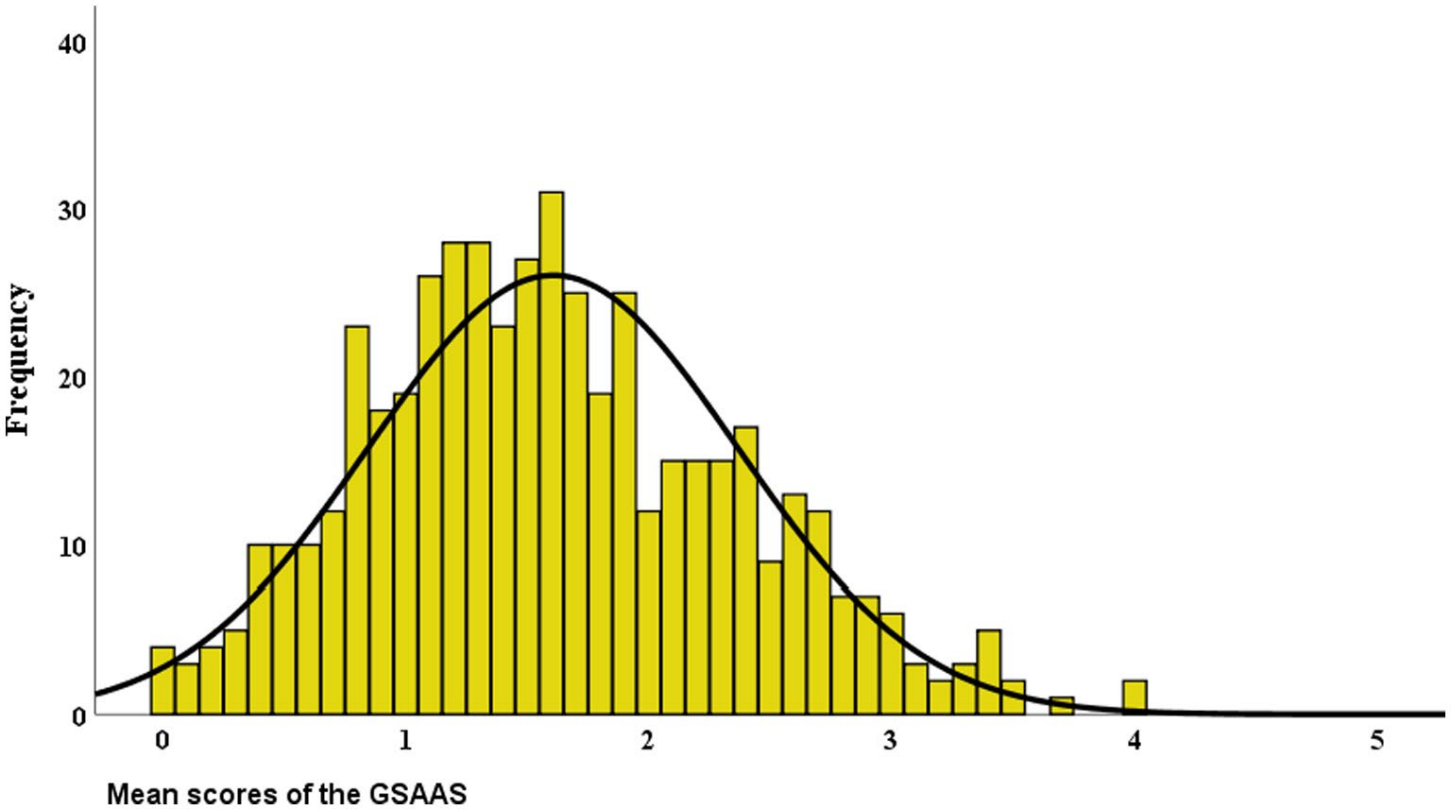
Frequency distribution of the mean scores of the GSAAS (*N* = 496).

Figure 2 shows the distribution of the mean scores of the GSAAS. With a skewness of 0.338 and a kurtosis of −0.148, the distribution of scores was approximately normal. The mean total score on the GSAAS was 1.61 (*SD* = 0.76) with a range from 0 to 4. Most scores were centered just below the midpoint of the scale. The descriptive statistics of the GSAAS, the SOC-13, the OQ-45, and the MHC-SF can be found in Table 5. This table also presents the correlations between the GSAAS and its validation measures.

**Table 5:** Means, standard deviations and correlations for outcome variables with the GSAAS.

	Mean (*SD*)	GSAAS
GSAAS (*N* = 496)	1.61 (0.759)	
SOC-13 (*N* = 488)	3.87 (0.933)	0.657^***^
MHC-SF (*N* = 496)		
Emotional well-being	2.46 (1.187)	0.568^***^
Social well-being	2.00 (1.103)	0.537^***^
Psychological well-being	2.38 (1.101)	0.655^***^
Total Well-being	2.26 (1.004)	0.662^***^
OQ-45 (*N* = 496)		
Symptomatic Distress	46.66 (20.12)	−0.698^***^
Interpersonal Relationships	10.48 (5.31)	−0.403^***^
Social Role	12.45 (5.57)	−0.386^***^
Total Psychopathology	50.50 (20.77)	−0.687^***^

There are variations in N due to missing data. ^***^p < 0.001.

In line with our expectations, there were strong positive correlations between the GSAAS and the SOC-13 (*r* = 0.657) and the GSAAS and the MHC-SF (*r* = 0.662) and its subscales PW (*r* = 0.665) and EW (*r* = 0.568) and SW (*r* = 0.537). As expected, a strong negative correlation was found between the GSAAS and the OQ-45 (*r* = −0.687) and its subscale OQ-SD (*r* = −0.698). Moderate correlations were found for the OQ-45 subscales OQ-IR (*r* = −0.403) and OQ-SR (*r* = −0.386).

Incremental validity was established using a hierarchical regression analysis (see Table 6). The GSAAS accounted for 7.4% additional variance in symptomatic distress (OQ-SD) beyond a Sense Of Coherence (SOC-13). This increase in explained variance was significant (*p* < 0.001).

**Table 6:** Hierarchical regression analysis: the GSAAS as predictor of SD (OQ-45) beyond SOC (SOC-13).

Step	Predictor	*B*	SE	*β*	*t*	Δ*R*^2^
1						0.567^***^
	SOC-13	−0.431	0.017	−0.753	−25.246^***^	
2						0.074^***^
	SOC-13	−0.295	0.021	−0.515	−14.296^***^	
	GSAAS	−0.271	0.027	−0.362	−10.034^***^	

^***^p < 0.001.

## 3. Discussion

The current study aimed to evaluate the GSAAS, a new measure to assess people’s generic sense of the ability to adapt, and to evaluate its psychometric properties in a Dutch sample of outpatient adults with mental health problems. The ability to adapt can be considered a vital aspect of health (Jambroes et al., 2016) and a process indicator for treatment (Bohlmeijer and Westerhof, 2021).

Overall, the results of our study suggest that the GSAAS factor structure was best represented by a single-factor model in both samples, with all items tapping into a single underlying construct. The GSAAS showed good internal consistency and convergent validity by demonstrating the expected relationships with theoretically related constructs, such as sense of coherence (Mittelmark et al., 2016), well-being (Taylor, 1983; Diener, 1984; Ryff, 1989; Stanton et al., 2007; van Leeuwen et al., 2012; Brüssow, 2013; Helgeson et al., 2014; Londono and McMillan, 2015; Audulv et al., 2016), and psychopathology (Eriksson and Lindström, 2007; Keyes et al., 2008; Grevenstein et al., 2016).

We found a strong relationship between the generic sense of ability to adapt (GSAAS) and a sense of coherence (SOC-13). Both instruments measure to what extent people might use their resources to overcome resistance and feel that stressors in their environment are manageable. Previous studies already proved the conceptually related construct of sense of coherence to be a strong predictor of psychopathology (Eriksson and Lindström, 2007; Grevenstein et al., 2016). The moderate to strong negative correlations between the GSAAS and symptomatic distress also confirm the convergent validity of the scale. In previous research into concepts strongly related to the ability to adapt, it has been conceptualized that lower experienced levels of ability to adapt will be associated with higher levels of anxiety (Roos et al., 2021) and depression (Cuijpers et al., 2021). Apart from the aforementioned theory about the sense of coherence, this also corresponds with theories about similar constructs such as self-management (Barlow et al., 2002; Lorig and Holman, 2003), psychological flexibility (Hayes, 2004; Kashdan and Rottenberg, 2010), sense of mastery (Bohlmeijer et al., 2003; Pinquart and Forstmeier, 2012), and identifying, describing, integrating, and regulating emotions (Bohlmeijer et al., 2003; Dunn, 2007; Pinquart and Forstmeier, 2012; Kramer, 2014). This underlines the importance further developing the more widely concept of the ability to adapt, and thus the application of a generic questionnaire as the GSAAS. Compared with the relations of the ability to adapt with psychopathology and symptomatic distress, the observed relations with family and friends and observed functioning in work and leisure were moderate and relatively low. One explanation for this lower correlation could be that relations and roles can be seen as *sources* of adaptation, where psychopathology and symptomatic distress are the *outcome*. This also fits into the model of sustainable mental health (Bohlmeijer and Westerhof, 2020, 2021).

An important finding of the current study is that the GSAAS accounted for 7.4% additional unique variance in symptomatic distress in comparison to the SOC-13 confirming its incremental validity. This finding shows the added value of the GSAAS. Where the SOC-13 focuses on powerlessness and environmental influence, the GSAAS focuses on decisiveness and the process of dealing with circumstances. In conclusion, with the GSAAS we now have an instrument with good psychometric properties to measure a generic sense of the ability to adapt. This may have several important implications. The scale could be used in clinical studies to assess the impact of psychological treatments on adaptation. The recently introduced theoretical model of sustainable mental health proposes the ability to adapt as a core indicator of recovery and resilience (Bohlmeijer and Westerhof, 2020, 2021). While distress and mental well-being may fluctuate across time depending on current life-events, a sense of the ability to adapt is an indicator whether a person is able to successfully cope with difficult life-events and restore good mental health. The availability of the GSAAS allows more empirical studies to validate the assumptions of the model of sustainable mental health. For example, it can be studied whether a sense of the ability to adapt indeed mediates the impact of dysfunctional and functional cognitions, emotions, and behavior on psychopathology and well-being.

The GSAAS can be implemented in clinical practice to validate the new sustainable mental health model to contribute to the recently initiated transition of focus from disease to health and thus stimulate a more balanced treatment in (mental) health care (Bohlmeijer and Westerhof, 2021). Treatments may focus on promoting adaptive regulations of difficult emotions or replacing or defusing from unrealistic and unhelpful cognitions. When these interventions are successful, a strengthening of the sense of the ability to adapt should be observed. In addition, interventions promoting personal resources should also enhance a sense of the ability to adapt. The GSAAS could thus be a practical, easy, and quick tool measuring a vital process of change in treatment.

A few limitations of the studies should be considered. First, in study 1, participants were mainly students with a high level of education, who possibly interpreted and evaluated their generic sense of ability to adapt in a specific way. Future research should study generalizability of the validity in other age- and education groups. However, in study 2 where the age range was wider and the level of education was mainly moderate, the GSAAS also demonstrated good psychometric properties. Also, across gender, age, and education, the factor structure showed sufficient measurement invariance. Second, any temporal directional nature of the relationship between the ability to adapt and psychopathology cannot be deduced from the results of the current cross-sectional study. Future longitudinal studies should also reveal if the GSAAS is sensitive to detect changes over time and can thus be used accurately as an evaluation tool in treatment settings. Likewise, in a follow-up study of psychometric qualities, adding a retest reliability study could substantiate the reliability of the GSAAS even more strongly. Furthermore, we suggest measurement invariance analyses to be applied in several psychiatric and somatic clinical and also non-clinical studies to investigate whether GSAAS scores can be meaningfully compared across clinical populations. Finally, in the present study, we only investigated incremental validity over sense of coherence. This was done because the sense of coherence is theoretically strongly related to adaptation and both can be considered as determinants or mediators of health. We found a strong association between well-being and the ability to adapt, as has been found earlier with the SOC (Getnet and Alem, 2019). In the future, it might be interesting to investigate the association between the quality of life and the ability to adapt, as the association with the SOC is also strong (Eriksson and Lindström, 2007).

## 4. Conclusion

All in all, the current study introduced the GSAAS as a valid and reliable tool, measuring the perceived ability to adapt. The unidimensional scale appears to be promising in assessing people’s generic sense of the ability to adapt and can be used in both (non-)clinical settings and research settings. The GSAAS shows its potential to contribute to the general understanding of mental health, where the concept of well-being did this before by leaving the one-sided psychopathology-oriented approach (Franken et al., 2018). Further research is recommended to establish stronger evidence for the psychometric properties of the scale in different target groups and its contribution to the evaluation and support of the model of Sustainable Mental Health in particular and the positive psychology framework in general (Bohlmeijer and Westerhof, 2020, 2021).

## Data availability statement

The raw data supporting the conclusions of this article will be made available by the authors, without undue reservation.

## Ethics statement

The studies involving human participants were reviewed and approved by Medical Ethics Review Committee (METC) from Twente University, under the Law on Research Involving Human Subjects (File numbers: 15092 and K18-19). The patients/participants provided their written informed consent to participate in this study.

## Author contributions

KF: first authorship, responsible for data processing and analysis, and display in manuscript. PS and KD: support and collect the data. PK: senior authorship and supporting data analysis. All authors contributed to the article and approved the submitted version.

## Conflict of interest

The authors declare that the research was conducted in the absence of any commercial or financial relationships that could be construed as a potential conflict of interest.

## Publisher’s note

All claims expressed in this article are solely those of the authors and do not necessarily represent those of their affiliated organizations, or those of the publisher, the editors and the reviewers. Any product that may be evaluated in this article, or claim that may be made by its manufacturer, is not guaranteed or endorsed by the publisher.
